# Breathin’ on the edge – the hidden complexity of pilot breathing regulators

**DOI:** 10.3389/fphys.2026.1745844

**Published:** 2026-02-17

**Authors:** Karoliina Messo, Heikki Mansikka

**Affiliations:** 1 Insta ILS, Tampere, Finland; 2 Department of Military Technology, National Defence University, Helsinki, Finland

**Keywords:** continued flow delivery, expiratory load, flow control, life support system (LSS), on-demand regulator, pilot-regulator interactions, safety-pressure system

## Abstract

The life support system of high-performance military aircraft is designed to protect aircrew from all adverse respiratory conditions. Many of its critical functions depend on an on-demand regulator, required to deliver breathing gas flow solely in response to pilot’s respiratory demands. However, the performance of the regulator when operated at the lower bound of, or outside, its specified operational range is not well known, understood, or fully characterized. To address this gap, we examined the performance of a CRU-103A/P safety pressure on-demand regulator when connected to a Gentex 5400 flight mask and supplied by a GGU-12/A on-board oxygen generating system–a life support system configuration employed in a variety of aircraft platforms. Regulator inlet pressures were controlled within and below the specified operating range (5–120 psig), specifically at 10, 6, 4, and 2 psig, producing a range of resting inspiratory flow demands in the participant. A dedicated, in-house–developed measurement system was used to capture high-resolution mask and regulator outlet pressures, as well as inspiratory and expiratory flows. Our results showed that an increase in peak inspiratory flow demand sufficient to produce mask pressures approximately 1 mbar or more below typical resting minimum values occurred at regulator inlet pressures below 10 psig. This led to continued regulator flow delivery during the early phase of expiration, resulting in elevated regulator outlet and mask pressures. This demonstrated that at regulator inlet pressures near or below the lower limit of the operational range, high inspiratory flow demand delayed regulator closure at the onset of expiration. Consequently, a brief period of continued breathing gas delivery occurred during early expiration. Further, the findings indicated that the user’s expiratory load increased at the onset of expiration due to a user-triggered flow termination failure in the regulator. This occurred as expiratory pressure propagated through the open inhalation valve to the regulator outlet and further to the rear of the mask exhalation valve, adding additional backpressure required to open it. The present study highlights the need for accurate, high-resolution monitoring of life support system’s performance. Such monitoring system would ensure that the life support system remains under the pilot’s control and imposes minimal respiratory loading. This is of vital importance for reducing dyspnea and allowing the pilot to allocate cognitive resources to mission-focused tasks, while also mitigating hypo- or hypercapnia that could compromise physiological normoxia.

## 1 Introduction

The aircrew of high-performance military aircraft must be provided with a life support system (LSS) that maintains physiological normoxia. A typical LSS consists of an onboard oxygen generating system (OBOGS), an on-demand regulator, and a flight mask. Ideally, the LSS fully protects aircrew against all potential adverse respiratory conditions and automatically adjusts to changing environmental conditions and emergency situations. Key operational characteristics that enable these functions include the provision of supplemental oxygen, adequate system flow capacity, minimal respiratory loading, and appropriate positive pressure breathing (PPB).

While the overall performance of an LSS depends on the integrated functioning of its individual components, many critical aspects are primarily determined by the design and performance of the on-demand regulator. The regulator governs breathing gas pressure and flow delivery to the pilot in accordance with environmental conditions and the pilot’s respiratory demands, respectively. Furthermore, it must operate effectively across the full range of inlet pressures that may occur within the LSS, while maintaining minimal breathing resistance.

The breathing gas supplied to the on-demand regulator by an OBOGS has two main characteristics. First, the product gas from the OBOGS is an air mixture enriched in both argon and oxygen. Second, the pressure at which an OBOGS supplies product gas is determined primarily by the pressure of the conditioned engine bleed air supplied to the OBOGS. This supply pressure can vary considerably between aircraft and operating conditions, depending, for example, on the engine’s power settings (Ernsting and Miller, 1996).

An essential element of the on-demand regulator is the pressure-balanced demand valve, which governs the delivery of breathing gas to the pilot. Its design ensures that the inlet pressure applies equal and opposing forces on the valve, thereby minimizing the effect of inlet pressure variations on valve opening. The demand valve is actuated by the differential pressure across a breathing diaphragm, a sensing element that responds to pressure changes. The on-demand regulator references the cabin pressure on one side of the diaphragm, while the other side senses the downstream pressure, i.e., the regulator outlet pressure. When the pilot is not inhaling, the pressure system is designed to remain balanced and closed. In a safety pressure system, a mechanical spring applies an additional constant force to the reference side of the diaphragm. Consequently, when the on-demand system is at rest, the regulator outlet pressure is maintained slightly above cabin pressure, a value referred to as the safety pressure. The nominal safety pressure is typically a few millibars. The presence of safety pressure in the flight mask ensures that any leakage from an imperfect mask seal results in outward flow. This outward flow prevents hypoxia by ensuring cabin air does not dilute the breathing gas. Positive pressure breathing (PPB) is achieved by pressure loading the diaphragm in accordance with the cabin altitude and G-forces. PPB is required to maintain adequate alveolar oxygen pressure in high altitude-flights and to withstand high-G maneuvers.

The flight mask used with an on-demand regulator providing safety pressure and PPB must include an exhalation valve, to which an equivalent backpressure is applied to preserve gas in the mask when it is delivered under pressure. The mask is also fitted with a non-return inhalation valve. The backpressure is taken from the regulator outlet near the inhalation valve inlet via a compensation tube integrated into the mask.

**FIGURE 1:**
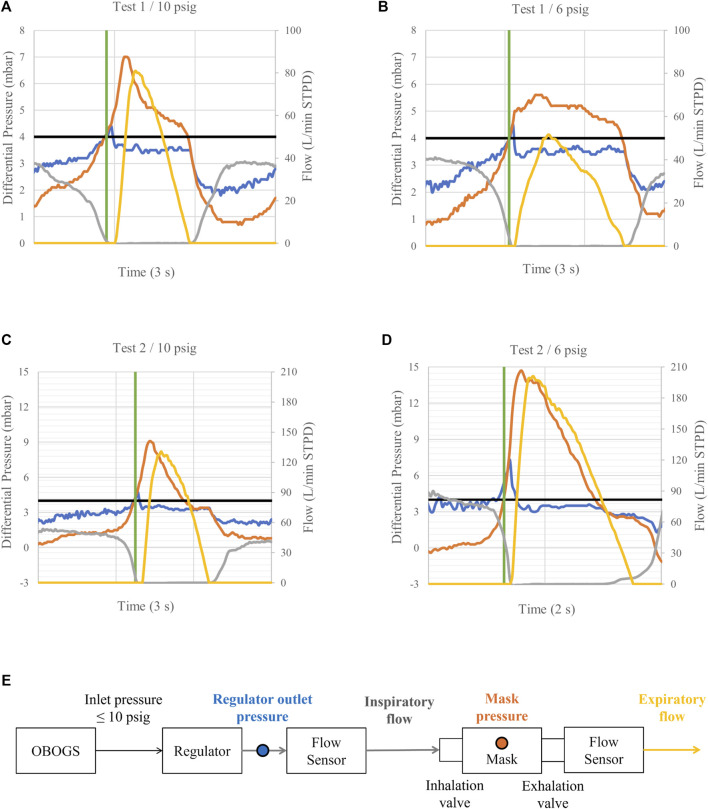
A sample of mask and regulator output pressures relative to ambient pressure, together with inspiratory and expiratory flows, measured at regulator inlet pressures of 10 **(A,C)** and 6 psig **(B,D)**. Expiratory flows are shown to provide a complete representation of breathing dynamics. Blue line: regulator outlet pressure. Orange line: mask pressure. Grey line: inspiratory flow. Yellow line: expiratory flow. Black line: nominal safety pressure. Green line: start of expiration. Inspiratory and expiratory phases are identified based on mask pressure, with inspiration occurring below and expiration above the nominal safety pressure. The measurement locations of the variables are shown in **(E)**. Note the decreased minimum mask pressure during inspiration at 6 psig in **(D)** compared to the reference condition in **(C)**. Observe also the continued regulator flow delivery and increased mask and regulator outlet pressure spikes during the early phase of expiration in **(D)** compared to **(C)**.

The designed interaction between the safety pressure on-demand regulator and the pilot can be understood by examining the variations in regulator outlet and mask pressures, as well as the inspiratory and expiratory flows, throughout a complete respiratory cycle, as illustrated in Figures 1A,C. For clarification, Figure 1E provides a schematic of the LSS with the measurement locations of the variables presented. At the start of expiration, shown by the green line, the safety pressure system is closed and balanced. The regulator outlet and mask pressures match the nominal safety pressure. With the flight mask inhalation and exhalation valves closed, there is no inspiratory or expiratory flow. Expiratory flow begins when the pilot slightly increases the mask pressure above the safety pressure, opening the mask exhalation valve. During expiration, the regulator outlet pressure remains close to the nominal safety pressure and is isolated from the mask pressure due to the closed inhalation valve. At the end of expiration, the safety pressure system returns to balance, and the exhalation valve closes. The pilot’s inspiratory flow demand creates a negative pressure inside the mask, opening the mask inhalation valve, which in turn reduces the regulator outlet pressure. This draws the diaphragm inward, opening the demand valve and allowing breathing gas to flow to the pilot. At the end of inspiration, the mask and regulator outlet pressures return to the nominal safety pressure, the inhalation valve closes, regulator flow ceases, and the safety pressure system returns to balance. An important feature of the on-demand valve is its linear pressure–flow characteristics, meaning that a given decrease in regulator outlet pressure produces a consistent inspiratory flow. This ensures that breathing gas is delivered at a rate and volume proportional to the pilot’s inspiratory demand.

The performance of the on-demand regulator depends on operating within its specified pressure and flow envelope. When inlet pressure falls below the minimum operating pressure or flow demand exceeds the regulator’s capacity, the diaphragm pressure balance can no longer be maintained because there is not enough available pressure to sustain normal operation. Under these conditions, even a pressure-balanced demand valve may exhibit instability, reduced flow capacity, or loss of control accuracy. This highlights the importance of maintaining regulator operation within its intended pressure and flow limits to ensure operational safety and system integrity. However, under engine idle power conditions, the pressure of the breathing gas supplied to the regulator inlet may be as low as 5 psig (pounds per square inch gauge, i.e., pressure relative to ambient atmospheric pressure) (Ernsting and Miller, 1996), corresponding to the lower bound of a typical on-demand regulator’s operating range.

Given the safety-critical function of the regulator, compromised performance may increase the pilot’s respiratory loading and thereby elevate the work of breathing. Elevated inspiratory or expiratory pressures can alter alveolar ventilation, potentially contributing to hypocapnia or hypercapnia (Connolly et al., 2021; Elliott and Schmitt, 2019; West, 2013; Ernsting and Miller, 1996). Both conditions are known to adversely affect cognitive function, underscoring the importance of maintaining regulator performance within its intended operating envelope to ensure pilot safety.

At present, there is no on-board system capable of monitoring regulator performance or overall pressure system integrity in real time to mitigate the potential risk of unexpected adverse respiratory conditions. Although recent studies have assessed pilots’ breathing performance during test flights using the VigilOX system (NASA Engineering and Safety Center, 2020), as well as in controlled laboratory settings (Robinson et al., 2022) and through simulation-based models (Ribeiro Rodrigues et al., 2024), there remains a lack of confirming or follow-up studies to validate these findings (Shaw and Harrell, 2023). Also, improved monitoring systems are needed to address the measurement limitations observed in previous studies (see, e.g., NASA Engineering and Safety Center, 2020). Consequently, the effects of compromised regulator internal pressure balance on the integrity of the safety pressure system are not well known, understood, or fully characterized. As each regulator design implements its internal pressure balance differently, the resulting effects are likely to depend on the specific regulator type employed.

This study focused on a safety pressure system comprising a CRU-103A/P on-demand regulator and a Gentex 5400 flight mask, a configuration used in a variety of aircraft platforms, including the F/A-18 and T-45. A dedicated, in-house–developed measurement system was employed to assess the safety pressure system’s integrity under both baseline and compromised regulator internal pressure balance, using reference and reduced inlet pressures, respectively. The CRU-103A/P is specified to operate at inlet pressures ranging from 5 to 120 psig (Naval Air Systems Command, 2017). A reference inlet pressure of 10 psig was chosen, representing the minimum pressure used during CRU-103A/P certification approval (Naval Air Systems Command, 2017). Under this reference condition, the system’s integrity is considered to be as designed, with the pressures and flows in the safety pressure system similar to those characterized in Figures 1A,C. The reduced inlet pressures were chosen as 6, 4, and 2 psig, representing levels expected to be intermediate (6 and 4 psig) or inadequate (2 psig) for sustaining normal regulator internal pressure balance. Each of these reduced inlet pressure conditions was compared with the reference condition to evaluate the integrity of the safety pressure system.

## 2 Materials and methods

### 2.1 Participants

This study evaluated the performance of a CRU-103A/P regulator under controlled extreme conditions rather than population-level physiological effects. A single healthy male test technician (57 years) was used to generate repeatable respiratory demand patterns, serving as a controlled simulator rather than a representative human subject. This approach allowed reproducible evaluation of the safety pressure system’s performance under specific operational stress conditions. The participant had no known history of respiratory or cardiovascular conditions. Written, informed consent was obtained prior to participation. Two tests were conducted in the study. The tests were administered by the first author. In Finland, the ethical evaluation of non-medical research involving human participants follows the guidelines established by the Finnish National Board on Research Integrity. Based on these guidelines, the research design of this study did not necessitate an ethical review statement from a human sciences ethics committee. All procedures were conducted in accordance with the Declaration of Helsinki.

### 2.2 Instrumentation and data collection

A GGU-12/A OBOGS (Eaton Aerospace, Davenport, IA, United States) was used to deliver compressed breathing gas to the inlet of a certified CRU-103A/P on-demand regulator (Eaton Aerospace, Davenport, IA, United States). The OBOGS supply pressure was controlled using a TTU-518A/E OBOGS test set (Eaton Aerospace, Davenport, IA, United States). A pressure indicator at the regulator inlet was used to adjust and maintain the regulator inlet pressure at the target levels. The oxygen concentration of the breathing gas depends on both the OBOGS supply pressure and the breathing gas flow, with the lowest concentrations occurring at low supply pressures and high breathing gas flows (Naval Air Systems Command, 2019). In this study, the OBOGS was supplied with compressed air, and the regulator provided a safety pressure of approximately 4.0 mbar. As a result, no hypoxic breathing gas was generated during the tests. Two 5400 model flight masks (Gentex, Zeeland, MI, United States) were used, one for each test. The purpose was to enable test repetition, not mask comparison. All LSS components were standard and equivalent to those currently in operational use by the Finnish Air Force.

A dedicated in-house–developed measurement system was used to collect data on mask pressure, regulator outlet pressure, and inspiratory and expiratory flows at a sampling rate of 50 Hz (Messo and Mansikka, 2025). One of the system’s two DLVR-series digital differential pressure sensors (Amphenol, St. Marys, PA, United States) was used to measure regulator outlet pressure via a commercial adapter with an integrated measurement port. The second sensor was used to measure mask pressure through a 3-mm diameter tubing inserted into the flight mask from the side. Prior to each data collection session, mask fitting was checked for any leakage. Inspiratory flow was measured using one of the system’s two digital flow sensors (SFM3200-AW, Sensirion, Stäfa, Switzerland) positioned between the regulator outlet and the mask hose. Two 3-cm sections of straight piping with matched inner diameter were used to minimize flow disturbances. An in-house–developed adapter was used to attach the second flow sensor to the mask exhalation valve for expiratory flow measurements.

Physiological and subjective data were captured solely to ensure that each data collection session was conducted safely. Heart rate (HR) and peripheral oxygen saturation (SpO2) were monitored using a fingertip pulse oximeter (ECO001, Esperanza, Germany). Physiological safety limits were set at a minimum SpO2 of 96% (Torp et al., 2023) and a maximum HR of 100 bpm (Henning and Krawiec, 2023). The participant was instructed to evaluate perceived breathlessness using the Modified Borg Dyspnea Scale (MBS) throughout each session. The MBS is a subjective 0 (no breathlessness) to 10 (maximal breathlessness) scale widely used in clinical settings (Crisafulli and Clini, 2010). The subjective termination criterion was set to an MBS score of 6, representing severe breathlessness. Scores of 5 were considered acceptable, given the short duration of the data collection sessions and continuous SpO2 monitoring. The participant was also allowed to terminate the session at any time if any other discomfort occurred.

### 2.3 Procedure

Two tests were conducted at the maintenance facilities of Insta ILS, Finland, with a 24-h interval between the tests. One test was conducted for each flight mask. For both tests, four data collection sessions were conducted. In each session, a regulator inlet pressure of 10, 6, 4, or 2 psig was used. The sessions were carried out in descending order, from 10 to 2 psig. Each session lasted 5 minutes, with 15–45 min between subsequent sessions. At regulator inlet pressures of 6, 4, and 2 psig, the number of breaths per session in Test 1 was 112, 113, and 113, respectively. In Test 2, 122 breaths were recorded per session at each pressure.

Prior to the data collection sessions, the participant was briefed on the MBS and reminded of the termination criteria. All sessions followed the same procedure, with the participant instructed to breathe naturally and the test administrator continuously monitoring SpO2 and HR. After completion of the session, the participant provided a single score on the MBS.

No physiological or subjective limits were reached during the data collection sessions. The participant did not wish to terminate any session early.

### 2.4 Statistical analysis

For statistical analysis, measured data from each data collection session were divided into segments and samples as follows. First, data segments were extracted from the measured data. An expiratory segment contained measured data for one of three variables: inspiratory flow, regulator outlet pressure, or mask pressure during an expiratory phase. An inspiratory segment contained measured data for one variable: mask pressure during an inspiratory phase. Inspiratory and expiratory phases were defined based on mask pressure, with inspiratory phases occurring when mask pressure was below the nominal safety pressure and expiratory phases when it was above it.

Then, segments were constructed into single samples, the smallest unit of analysis. Expiratory segments were constructed into early-phase samples representing the first 300 ms of each segment. Inspiratory segments were constructed into samples corresponding to the minimum value within each segment.

Statistical analyses were performed using IBM SPSS Statistics, version 30. For each variable, mean values were computed across the corresponding samples within each data collection session. Given the continuous nature of the variables and the relatively large sample sizes (n > 100), paired samples t-tests were performed to compare reduced regulator inlet pressure conditions with the 10-psig reference condition. All tests were conducted with an alpha level of 0.05. Effect sizes were calculated using Cohen’s d.

## 3 Results

Results are summarized in Tables 1–4, with significant differences interpreted as evidence of systematic changes in flow or pressures across reduced regulator inlet pressure conditions. Reductions in inlet pressure generally produced statistically significant changes in the variables relative to the reference condition. The 2 psig inlet pressure conditions were associated with the greatest increases in mean values (Tables 1–3) and the lowest minimum values (Table 4). In Tables 1–3, “Mean Diff” refers to the average difference in the analyzed variable between each reduced inlet pressure condition and the 10-psig reference condition, calculated on a breath-by-breath paired basis during the early phase of expiration.

**TABLE 1:** Paired-samples t-test results for inspiratory flow during the early phase of expiration for reduced regulator inlet pressures.

Comparison	*n*	Mean diff	*t*	Df	*p*	Cohen’s *d*
Test 1: 6 psig <10 psig	112	0.172	3.59	111	<0.001	0.34
Test 1: 4 psig >10 psig	113	0.323	7.47	112	<0.001	0.70
Test 1: 2 psig >10 psig	113	8.405	21.9	112	<0.001	2.06
Test 2: 6 psig >10 psig	122	2.412	13.2	121	<0.001	1.20
Test 2: 4 psig >10 psig	122	4.857	15.4	121	<0.001	1.39
Test 2: 2 psig >10 psig	122	9.488	32.1	121	<0.001	2.91

**TABLE 2:** Paired-samples t-test results for regulator outlet pressure during the early phase of expiration for reduced regulator inlet pressures.

Comparison	*n*	Mean diff	*t*	Df	*p*	Cohen’s *d*
Test 1: 6 psig <10 psig	112	−0.016	−2.70	111	<0.01	−0.26
Test 1: 4 psig <10 psig	113	−0.078	−11.00	112	<0.001	−0.99
Test 1: 2 psig >10 psig	113	1.074	14.30	112	<0.001	1.35
Test 2: 6 psig >10 psig	122	0.162	7.70	121	<0.001	0.70
Test 2: 4 psig >10 psig	122	0.511	7.76	121	<0.001	0.70
Test 2: 2 psig >10 psig	122	1.502	17.00	121	<0.001	1.54

**TABLE 3:** Paired-samples t-test results for mask pressure during the early phase of expiration for reduced regulator inlet pressures.

Comparison	*n*	Mean diff	*t*	Df	*p*	Cohen’s *d*
Test 1: 2 psig >10 psig	113	0.581	8.01	112	<0.001	0.75
Test 2: 6 psig >10 psig	122	0.110	3.09	121	<0.01	0.28
Test 2: 4 psig >10 psig	122	0.215	9.12	121	<0.001	0.83
Test 2: 2 psig >10 psig	122	0.448	15.70	121	<0.001	1.42

**TABLE 4:** Paired-samples t-test results for minimum mask pressure during the inspiratory phase for reduced regulator inlet pressures.

Comparison	Mean (cond.)	Mean (ref. cond.)	*t*	Df	*p*	Cohen’s *d*
Test 1: 6 psig >10 psig	0.64 ± 0.28	0.56 ± 0.24	2.15	112	<0.05	0.20
Test 1: 4 psig >10 psig	0.70 ± 0.21	0.56 ± 0.24	4.53	112	<0.001	0.43
Test 1: 2 psig <10 psig	−1.33 ± 0.87	0.57 ± 0.24	−22.90	113	<0.001	2.14
Test 2: 6 psig <10 psig	−0.64 ± 1.21	0.27 ± 0.27	−8.28	122	<0.001	−0.75
Test 2: 4 psig <10 psig	−0.68 ± 0.72	0.27 ± 0.27	−13.91	122	<0.001	1.25
Test 2: 2 psig <10 psig	−1.32 ± 0.87	0.57 ± 0.24	−22.90	113	<0.001	2.14

During the early phase of expiration, regulator continued flow delivery increased markedly as inlet pressure decreased. All reduced inlet pressure conditions, except one at 6 psig, produced significantly greater inspiratory flow compared with the reference condition (Table 1).

The regulator outlet pressure showed a similar pattern during the early phase of expiration (Table 2). A notable increase in outlet pressure was observed when the inlet pressure was reduced to 2 psig. At the intermediate inlet pressures of 6 and 4 psig, mixed responses were observed with either smaller increases or slightly lower outlet pressures compared with the reference condition. Analyses of mask pressure during the early phase of expiration (Table 3) confirmed this pattern, with all reduced inlet pressure conditions exhibiting significantly higher mask pressures than the reference condition.

During inspiration, the minimum mask pressure, reflecting the peak inspiratory flow demand, decreased sharply when the inlet pressure was reduced to 2 psig (Table 4). At the intermediate inlet pressures of 6 and 4 psig, a mixed response was observed, with the minimum mask pressures showing either significant decreases or slight increases compared with the reference condition.

Figures 1A–D compare the reduced regulator inlet pressure condition at 6 psig with the reference condition. Reference conditions are shown in Figures 1A,C. Mixed responses from the two tests are shown in Figures 1B,D. In Figure 1B, no increase in regulator continued flow delivery, nor any notable change in regulator outlet pressure, is observed during the early phase of expiration compared with the reference condition in Figure 1A. Furthermore, no notable difference in peak inspiratory flow demand is observed compared with the reference condition. In Figure 1D, by contrast, regulator continued flow delivery is observed to increase markedly, accompanied by elevated regulator outlet and mask pressures during the early phase of expiration, compared with the reference condition in Figure 1C. In addition, the peak inspiratory flow demand is observed to be notably higher compared with the reference condition.

## 4 Discussion

When inspiratory gas is flowing, the pressure at the regulator outlet is higher than the pressure in the mask. Under proper regulator function, the inspiratory flow ceases at end-inspiration, before the start of expiration, as the mask pressure reaches the nominal safety pressure. The regulator outlet pressure remains stable at this level throughout the expiratory phase, allowing the mask pressure to exceed it and open the mask exhalation valve. Contrary to previous findings (Robinson et al., 2022), we found that the regulator continued to deliver flow during the early phase of expiration at reduced regulator inlet pressures (Table 1), implying that the mask inhalation valve did not close before expiration began. This indicated that the regulator outlet pressure was incorrectly elevated above the mask pressure during this phase. It suggests that the regulator’s internal pressure balance was disrupted, compromising the linear pressure–flow characteristics of the regulator. As discussed later, this loss of linearity will have adverse effects on breathing dynamics, as pressures are communicated and gas flows without the user’s control.

However, regulator continued flow delivery was not an inevitable consequence of reduced regulator inlet pressure. In Test 1, compared with the 2-psig inlet pressure condition, both 6 and 4 psig inlet pressures resulted in similarly small changes in inspiratory flow, indicating that substantial changes occurred only at the very low inlet pressure of 2 psig (Table 1). Instead, under conditions of reduced regulator inlet pressure, regulator continued flow delivery during the early phase of expiration was strongly associated with the user’s peak inspiratory flow demand. Our results showed that inspiratory flow occurred only when the peak inspiratory flow demand increased substantially from the reference condition (Table 4). Figure 1 highlights this finding. Comparing the 6 psig inlet pressure conditions for the two tests, continued regulator flow delivery was observed only in Test 2, where the minimum mask pressure during inspiration was markedly decreased compared to the reference condition.

Earlier studies have shown that at regulator inlet pressures of 6 psig and below, regulator function is compromised, resulting in increasingly restricted flow delivery (Robinson et al., 2022). During natural breathing, Robinson et al. (2022) found that the MIL-STD-3050 requirements (Department of Defense, 2015) were marginally met at 6 and 4 psig, if at all, and were not met at 2 psig. At reduced inlet pressures, a substantially large peak inspiratory flow demand is therefore expected to exceed the flow capacity of the regulator. With the regulator operating under compromised internal pressure balance, the continued flow delivery beyond the inspiratory phase that we observed strongly suggests a cause–effect relationship between the altered pressure balance and the regulator’s failure to terminate flow appropriately. A comparison based on the differences in inspiratory flow during the early phase of expiration suggests that flow increased substantially as regulator inlet pressure decreased (Table 1). This finding supports the presence of flow-restricting effects in the regulator. It also highlights the user–regulator interaction, whereby the user is increasingly able to trigger continued regulator flow delivery during the early phase of expiration as the regulator’s flow capacity decreases. This occurs because the flow limit is reached even with progressively smaller peak inspiratory flow demands. A user-triggered flow termination failure in the regulator could pose a serious safety risk. When the regulator forces inspiratory gas into the mask during expiration, two possible outcomes may occur depending on the breathing dynamics. If expiration is not initiated, i.e., a breath-hold follows inspiration, the mask exhalation valve could be forced open, leading to simultaneous inspiratory and expiratory flow, a so-called flow-through event. If expiration follows inspiration, the user is forced to breathe against the forced inspiratory flow. As discussed later, this increases the user’s expiratory load.

Our finding of continued flow delivery beyond the inspiratory phase, resulting from demands that exceed the regulator’s flow capacity, is also supported by results reported for the Anti-G Straining Maneuver (AGSM) breathing technique (Messo and Mansikka, 2025). Despite being supplied with high pressure, the regulator consistently delivered flow in the absence of inspiratory demand whenever its specified flow capacity of 240 L/min was exceeded. This forced inspiratory flow subsequently forced the exhalation valve open when inspiration was followed by a breath-hold, as occurred during AGSM breathing, causing a flow-through event.

During inspiratory flow, whether occurring during inspiration or expiration, the exhalation valve remains closed because the regulator outlet pressure exceeds the mask pressure. Consequently, when flow is delivered during expiration, expiratory pressure must be transmitted to the regulator outlet through the open inhalation valve. Our results indicated that regulator outlet pressures increased significantly during the early phase of expiration (Table 2). Previous studies have attributed these regulator outlet pressure spikes to regulator “overshoots” (Robinson et al., 2022). The present findings, however, indicated that the spikes were triggered by the user’s expiratory effort.

Continued flow delivery during the early phase of expiration increases the user’s expiratory load. The transmitted expiratory pressure to the regulator outlet propagates through the mask compensation tube and adds additional backforce to the exhalation valve. An equivalent increase in expiratory pressure is therefore required to open the exhalation valve. As shown in Table 3, mask pressures increased significantly during the early phase of expiration. While earlier work has explained this increased exhalation valve backforce as the result of regulator outlet pressure “overshoots” being propagated through the mask compensation tube (Robinson et al., 2022), our findings point instead to expiratory pressure being transmitted through the same pathway.

Figure 1 demonstrates the effects of continued flow delivery during the early phase of expiration on regulator outlet and mask pressures. When comparing the pressures for Test 2 in Figures 1C,D, it can be seen that both pressures increased markedly. In Test 1, where no continued flow delivery occurred, the regulator outlet pressure remained isolated from the mask pressure. This is indicated by the negligible differences in regulator outlet pressures when comparing Figures 1A,B. A comparison of the differences in inspiratory flow, regulator outlet pressure, and mask pressure during the early phase of expiration suggests that pressures increased with increasing flow (Tables 1–3). This is expected, as higher flow is inherently associated with a more pronounced elevation in regulator outlet pressure, necessitating a higher mask pressure to cease inspiratory flow and to open the exhalation valve. This effect is likely further amplified by the increasing backforce applied to the exhalation valve.

### 4.1 Impact of regulator complexity on respiratory dynamics

The regulator and mask constitute components of a complex, pressure-balanced system. Valve sequencing and actuation are designed to be controlled solely by the user’s respiratory demands. Consequently, the user functions as the sole flow trigger, with opening and closing of the valves synchronized to changes in mask pressure, which determine the inspiratory and expiratory phases. However, our findings demonstrated that the regulator, despite its seemingly straightforward design, is a far more complex pressure–flow component than is apparent from current functional testing protocols (Naval Air Systems Command, 2017). Our results revealed adverse effects on flow control and respiratory loading even in a system composed of functionally approved components operating within specified environmental conditions.

The results of this paper support the conclusion of Shaw and Harrell (2023) that more accurate and high-resolution test devices and on-board monitoring systems are needed. Both flow and pressure must be captured with sufficient resolution to ensure the integrity of the safety pressure system. Insufficient measurement resolution could easily miss important phenomena, such as continued flow delivery during the early phase of expiration, which are essential for fully explaining the regulator’s safety-critical functioning.

Our findings demonstrated that under conditions of compromised regulator performance, the user may inadvertently trigger adverse user–regulator interactions. This appears to occur when the regulator flow limit is reached, leading to excessive expiratory load. This is a concerning outcome for several reasons. First, the human respiratory system possesses compensatory reflexes to counteract increased respiratory loading. It continuously alters the within-breath dynamics to minimize the work of breathing while maintaining the required alveolar ventilation (Napoli et al., 2022). However, reaching the limit of breathing compensation may place the pilot at risk of hypocapnia or hypercapnia (Connolly et al., 2021; Elliott and Schmitt, 2019; West, 2013; Ernsting and Miller, 1996). Early identification of increased respiratory loading, prior to the engagement of compensatory mechanisms, is therefore critical for mitigating adverse physiological effects and maintaining adequate alveolar ventilation (Bimal et al., 2025). Second, the pilot’s inspiratory flow demand can vary substantially across different phases of flight and among individual pilots (Robinson et al., 2022; NASA Engineering and Safety Center, 2020; Ernsting and Miller, 1996). This variability induces a highly variable expiratory load. Consequently, the pilot is required to continuously adjust breathing dynamics to compensate, contributing to increased respiratory effort.

The complexity of the regulator adds a counteractive element to the protective function of the LSS. Instead of reliably meeting the rate and volume of the inspiratory demand, the LSS may itself begin to “breathe” against the pilot. Despite providing altitude protection, the LSS may paradoxically induce hypoxia-like events (Connolly et al., 2021; Phillips, 2019; West, 2013; Davis et al., 2008) under unexpected operational conditions, potentially leading to a compromised respiratory state and impaired pilot cognitive performance. Our findings underscore the potentially serious consequences that may arise during operational flights when the performance of a single LSS component, and its interactions within a common LSS configuration, are not known, understood, or fully characterized.

### 4.2 Limitations and future directions

Despite providing novel and complementary insights into the interactions among the regulator, mask, and user, this study did not characterize regulator performance as part of a statistical population study. Instead, it focused on regulator performance characteristics under off-nominal and boundary operating pressure conditions, where performance is least understood and most operationally consequential.

The number of data-collection sessions was intentionally limited, as the primary objective was to identify failure modes rather than to estimate population-level performance metrics. In conjunction with prior observations reported in the literature, the present data capture a meaningful range of inspiratory flow demands and reveal complex and non-intuitive regulator responses.

While the results are in line with existing literature, the observed effect sizes (Cohen’s d; see Tables 1–4) are small and should be interpreted with caution when evaluating their operational relevance. It is plausible that the magnitude of these effects may change, and potentially decrease, when additional subjects or repeated experimental runs are included, which could further affect their practical significance. Nevertheless, presented findings underscore the need for future investigations that systematically identify failure modes across broader operational contexts, user respiratory demands, and LSS configurations. Such efforts would provide a framework for defining and computing appropriate failure quantification metrics, thereby offering context on their operational relevance. Additionally, establishing a scoring system for regulator performance would not only facilitate comparisons between individual units but also contribute to the development of future functional testing protocols and test devices, as well as aircraft warning systems. In particular, future work could evaluate the effects of observed failure modes on pilot physiology and cognitive performance. Such work would represent an important contribution to the mitigation of physiological events in real flight scenarios.

It should be noted that an increased expiratory pressure may negatively impact pilots’ G-tolerance. Although the extent of such effects is beyond the scope of the present study, changes to breathing pressure characteristics should be approached with caution and carefully evaluated.

Further, our study did not consider PPB conditions. Under PPB, regulator operation becomes even more complex, potentially masking fundamental interactions that we identified in a non-challenging performance environment. Future research is encouraged to progressively add complexity to the performance environment used in testing. Equally important, AGSM breathing should be incorporated to investigate regulator flow triggers of high magnitude and rapid onset.

## 5 Conclusion

This study characterized the performance of a CRU-103A/P regulator during natural breathing under reduced inlet pressures. We found that substantially large peak inspiratory flow demands resulted in continued regulator flow delivery during the early phase of expiration. The delayed cessation of flow added additional backforce to the exhalation valve. This occurred when the user pressurized the regulator outlet through the open inhalation valve at the onset of expiration. Both regulator outlet and mask pressures increased during the early phase of expiration. The results reveal the inherent complexity of the pilot’s regulator. Critically, this complexity can cause the LSS to interact with the pilot in a counterproductive manner–instead of supporting the pilot’s breathing demands, it can force the pilot to “breathe” against the LSS, potentially degrading the pilot’s cognitive state and compromising mission success and flight safety.

## Data Availability

The raw data supporting the conclusions of this article will be made available by the authors, without undue reservation.
